# Generation of Eosinophils from Cryopreserved Murine Bone Marrow Cells

**DOI:** 10.1371/journal.pone.0116141

**Published:** 2014-12-31

**Authors:** Kaila L. Schollaert, Michael R. Stephens, Jerilyn K. Gray, Patricia C. Fulkerson

**Affiliations:** Division of Allergy and Immunology, Department of Pediatrics, University of Cincinnati College of Medicine, Cincinnati Children’s Hospital Medical Center, Cincinnati, Ohio, United States of America; Universidade Federal de Juiz de Fora, Brazil

## Abstract

Eosinophils are produced in the bone marrow from CD34^+^ eosinophil lineage–committed progenitors, whose levels in the bone marrow are elevated in a variety of human diseases. These findings suggest that increased eosinophil lineage–committed progenitor production is an important process in disease-associated eosinophilia. The pathways central to the biology of the eosinophil lineage–committed progenitor remain largely unknown. Thus, developing new methods to investigate the regulators of eosinophil lineage–committed progenitor differentiation is needed to identify potential therapeutic targets to specifically inhibit eosinophil production. We tested cytokine regimens to optimize liquid cultures for the study of eosinophil lineage–committed progenitor and eosinophil precursor differentiation into mature eosinophils. Stem cell factor (but not fms-related tyrosine kinase 3 ligand) was required for optimal yield of eosinophils. Furthermore, we evaluated the effects of cell preservation and scale on the culture, successfully culturing functional eosinophils from fresh and frozen murine bone marrow cells and in a standard-sized and 96-well culture format. In summary, we have developed an adaptable culture system that yields functionally competent eosinophils from murine low-density bone marrow cells and whose cytokine regime includes expansion of progenitors with stem cell factor alone with subsequent differentiation with interleukin 5.

## Introduction

The bone marrow responds to systemic infection and inflammation with heightened hematopoiesis to replenish immune cells in a host-protective manner [1]–[3]. Cytokines and other inflammatory mediators have been shown to bias hematopoiesis to enhance production of specific effector cells, including granulocytes [4], [5]. Elevated numbers of blood and tissue eosinophils occur in numerous infectious and inflammatory diseases, and studies have demonstrated a critical role for the cytokine interleukin (IL) 5 in disease-associated eosinophilia [6]–[9]. Yet, the molecular regulators of IL-5–induced differentiation of the eosinophil lineage–committed progenitor (EoP) into mature eosinophils are under-investigated, likely due to the relative rarity of EoPs in the bone marrow under homeostatic conditions (0.05% of lineage-negative CD34^+^ progenitors) [10]–[12]. Although IL-5–targeted therapy is very effective in reducing mature eosinophil counts in the blood and bone marrow, the number of EoPs and the bone marrow’s capacity to produce eosinophils is unchanged [13]. As atopy, helminth infections and allergen challenge have all been shown to increase EoPs (CD34^+^CD125^+^ cells) in the bone marrow [14], [15], these findings highlight the need to identify novel targets specific to EoPs to potentially suppress eosinophil production by the bone marrow. However, the pathways central to the biology of the EoP, particularly those related to survival, expansion and differentiation, remain largely unknown. Therefore, a great demand exists for investigations focused on the rare EoP population and methods by which to conduct these investigations, especially given recent advances in EoP identification.

The plethora of transgenic and gene-disrupted mice available and the capability to now identify the EoP by surface markers have led to the current, unprecedented opportunity to culture hematopoietic progenitors explanted from these animals to study the regulation of EoP differentiation into mature eosinophils. A number of methods have been developed for the isolation and *ex vivo* expansion of rare bone marrow progenitor populations. The low-density bone marrow fraction (LDBM) of whole bone marrow (WBM) is known to be enriched with progenitors and has been used alone, and in conjunction with fluorescence-activated cell sorting, for isolation of distinct progenitor and precursor populations [16], [17]. A variety of cytokines and growth factor combinations have been used to expand progenitors *ex vivo*, with stem cell factor (SCF) and fms-like tyrosine kinase 3 ligand (FLT3L) demonstrating positive effects on CD34^+^ progenitors [18], [19]. However, the optimal conditions that result in functional eosinophils are unknown.

A high-yield *ex vivo* eosinophil culture system that results in phenotypically mature eosinophils has been developed [20]. Starting with unselected WBM and expanding progenitors with SCF and FLT3L prior to IL-5–mediated differentiation, this culture system has been highly useful and effective for studies focused on the function of mature eosinophils, as well as for evaluating the eosinophil production potential of bone marrow of different genetic backgrounds [21]–[26]. We investigated the optimal protocol for studying eosinophil differentiation, comparing iterations of cytokine regimens to maximize yield of functionally mature eosinophils while decreasing potential influence of other mature cells in the cultures. In addition, we evaluated the effects of cell preservation and scale, establishing methods to culture eosinophils from fresh and frozen bone marrow cells and in a standard-sized and 96-well format to allow for the greatest flexibility for future studies, including those that will investigate novel, EoP-specific targets to potentially suppress eosinophil production by the bone marrow.

## Materials and Methods

### Mice

In-house-bred, four- to eight-week-old, male and female wild-type BALB/c or CCR3-deficient [27] mice were used as the source of bone marrow cells. All mice were housed under specific pathogen-free conditions and handled, including euthanasia via carbon dioxide inhalation, under approved protocols (#2E09072) of the Institutional Animal Care and Use Committee of Cincinnati Children’s Hospital Medical Center.

### LDBM cell isolation

The femurs and tibiae of mice were crushed in a sterile mortar and pestle containing 1X phosphate-buffered saline (PBS) with 2% fetal bovine serum (FBS) and characterized (HyClone, Hudson, NH), and cells were collected by filtration through a 70-µm strainer. Red blood cells (RBCs) were lysed using RBC lysis buffer (Sigma-Aldrich, St. Louis, MO), and the remaining cells (0.5–1.5×10^8^ cells per 3 mL of 1X PBS) were subjected to centrifugation at 400×g for 30 minutes at room temperature on a Histopaque 1083 (Sigma-Aldrich) gradient. Deceleration was performed without brake. LDBM cells were aspirated from the interface and washed twice by resuspending with 25 mL 1X PBS and centrifuging 5 minutes at 500×g at room temperature. LDBM cells were then resuspended in 25 mL 1X PBS, and viability was determined by Trypan blue exclusion. For some experiments, WBM and LDBM cells were resuspended in characterized FBS (HyClone) and 10% dimethyl sulfoxide (DMSO; Sigma-Aldrich, St. Louis, MO), transferred to cryogenic vials and immersed in a room-temperature isopropanol bath prior to storing at −80°C. Cells were thawed in a 37°C water bath for approximately 1–2 minutes and washed in a 10-fold volume excess of 1X PBS.

### Liquid eosinophil cultures

LDBM cells were cultured at 0.8–1×10^6^ cells per mL in 6-well plates or 24-well plates containing Complete Media composed of Iscove's Modified Dulbecco's ***Media*** (IMDM) with Glutamax (Life Technologies, Grand Island, NY); 10% FBS, characterized (HyClone); 2 mM L-glutamine (Life Technologies); 50 µM β-mercaptoethanol (Sigma-Aldrich) and 1X penicillin and streptomycin (Life Technologies) and supplemented with SCF (Peprotech, Rocky Hill, NJ) and/or FLT3L (Peprotech) at the indicated doses. On Day 4, the media in each non-centrifuged plate was replaced with fresh Complete Media containing murine IL-5 (Peprotech) at the indicated doses. Media with IL-5 was changed every 2 days thereafter. Morphology of native and cultured eosinophils was evaluated by staining cytospins with Differential Quik Modified Giemsa stain (Electron Microscopy Sciences, Hatfield, PA). Imaging was performed using an Olympus BX51 microscope under oil immersion at 1000X magnification with an Olympus DP72 digital camera. For some experiments, LDBM cultures were plated in 96-well, round- and flat-bottom dishes at a density of 0.1–2×10^6^ cells per mL in 100 µL per well. Cells were allowed to settle undisturbed for 5 minutes prior to media aspiration to minimize cell loss during media changes. At the end of the culture period, cells were stained with FITC-CCR3 (83101, R&D Systems, Minneapolis, MN), PE-Siglec-F (E50-2440, BD Biosciences, San Diego, CA) and Live/Dead near-IR fixable stain (Molecular Probes, Grand Island, NY).

### EoP identification via flow cytometry

WBM or LDBM cells were washed twice in flow buffer (1X PBS containing 1 mM [ethylenediaminetetraacetic acid] EDTA) and suspended at a concentration of 10–100×10^6^ cells per mL flow buffer. Blocking was performed with 0.5–5.0 µg rat anti-mouse CD16/CD32 (BD Biosciences) for 5 minutes at 4°C. Cells were incubated with antibodies for 15–30 minutes at 24°C and then stained for 5 minutes with Live/Dead near-IR fixable stain (Molecular Probes). Cells were then washed twice and suspended in flow buffer. Antibodies used include: rat anti-mouse CD34-FITC (RAM34, BD Biosciences), rat anti-mouse CD125-PE (T-21, BD Bioscences), rat anti-mouse CD117-Brilliant Violet 421 (2B8, Biolegend, San Diego, CA), rat anti-mouse Gr-1 (Ly6G/C)-PE-Cy5 (RB6-8C5, Biolegend), rat anti-mouse CD3-PE-Cy5 (17A2, BD Biosciences), rat anti-mouse CD4-PE-Cy5.5 (RM4-5, Molecular Probes), rat anti-mouse CD8a-PE-Cy5.5 (5H10, Molecular Probes), rat anti-mouse CD19-PE-Cy5.5 (6D5, Molecular Probes) and rat anti-mouse CD45R (B220)-PE-Cy5.5 (RA3-6B2, Molecular Probes), as well as appropriate isotype controls. Heat-killed WBM (1 minute at 95°C), and live WBM were used as controls for live/dead staining. Data were acquired using LSR II, FACS Canto and LSR Fortessa flow cytometers (BD Biosciences) maintained by the Research Flow Cytometry Core at Cincinnati Children’s Hospital Medical Center.

### Flow cytometry analysis

Data were compensated and analyzed using FlowJo software (Tree Star, Ashland, OR). Compensation was calculated in FlowJo using unstained and stained cells (Live/Dead) and OneComp eBeads (eBioscience, San Diego, CA). Gate boundaries and shapes were determined using fluorescence-minus-one (FMO) controls including appropriate isotype antibodies.

### Gene expression

RNA was collected in TriPure reagent (Roche Diagnostics, Indianapolis, IN) and purified using the Direct-Zol RNA purification kit (Zymo Research, Irvine, CA) with in-column DNAse digestion. RNA concentrations were determined by NanoDrop (Thermo Scientific, Waltham, MA). A total of 1 µg cDNA was synthesized using iScript (Bio-Rad, Hercules, CA), and 10 ng was used as template for quantitative PCR on an ABI7900HT thermal cycler (Applied Biosystems, Grand Island, NY) using FastStart SYBR Green Master mix (Roche Diagnostics). Relative expression to *Gapdh* was determined by the 2-ΔΔCt method.

### Cytokine production

Cultured eosinophils were collected at Day 14, replated at 2.5×10^6^ cells per well in 1 mL in a 24-well tissue culture plate in Complete Media with murine IL-5 (Peprotech). Cell-free supernatants were harvested after 24 hours, and cytokine levels were determined by enzyme-linked immunosorbent assay per manufacturer’s instructions.

### Chemotaxis

Cell chemotaxis was evaluated using 96-well transwell plates (5.0-µM pore size, polycarbonate membrane; Corning, Tewksbury, MA) in a chemotaxis buffer composed of Roswell Park Memorial Institute medium (RPMI; RPMI 1640, no phenol red; Life Technologies) with added 0.5% bovine serum albumin (BSA) with low endotoxin (Sigma-Aldrich). Cultured eosinophils (6.25×10^5^ eosinophils per mL in 80 µL chemotaxis buffer) were placed in the upper well, and chemoattractants (CCL11/eotaxin-1 [Peprotech] or leukotriene B4 [LTB4; Cayman Chemical, Ann Arbor, MI]) in chemotaxis buffer (235 µL) at indicated concentrations were placed in the bottom well. In addition, a standard curve was generated with serial dilutions of cells, starting at 2×10^5^ cells per well, plated below the transwell in chemotaxis buffer. After a two-hour incubation at 37°C, the upper transwell insert was removed, and a cell viability dye (25 µL of WST-1; Clontech, Mountain View, CA) was added to each well. After an additional hour of incubation at 37°C, the absorbance at 420 nm was measured on a Synergy2 microplate reader (Biotek, Winooski, VT), and the total cell number in the bottom well was determined using the generated standard curve. Each condition was performed in triplicate and reported as the mean (± standard deviation [SD]) of each triplicate.

### Eosinophil peroxidase (EPO) activity assay

Cultured eosinophils were suspended in RPMI 1640, no phenol red (Life Technologies) containing 0.5% endotoxin-free BSA (Sigma-Aldrich, St. Louis, MO) at 1×10^6^ cells per mL, and a volume of 0.1 mL per well was transferred to a 96-well plate. Cells were incubated at 37°C for 30 minutes, and then phorbol 12-myristate 13-acetate (PMA, Sigma-Aldrich) was added at a concentration of 100 or 500 ng per mL and incubated for an additional 2 hours. OMB substrate buffer (0.05 M) was freshly prepared for each assay and contained 0.2 mg per mL o-phenylenediamine, 0.0243 M citric acid, 0.0514 M dibasic sodium phosphate, and 0.00012% H_2_O_2_ (Sigma-Aldrich). OMB substrate buffer (100 µL) was added to each well, and supernatants were incubated at room temperature for 10–15 minutes. As a positive control for the assay, total EPO activity of unstimulated cells (no PMA) was assayed as above with the addition of 0.01% Triton X-100 (Thermo Scientific). The colorimetric reaction was stopped with the addition of 50 µL per well of 2 N H_2_SO_4_, and absorbance (492 nm) was measured on a Synergy 2 plate reader. Data are presented as the fold change (mean ± SEM, *n* = 4 independent experiments with 3 wells per condition per experiment) in EPO activity detected in the supernatants of stimulated cells compared to unstimulated cells on the same plate.

### Actin polymerization assay

Native eosinophils were collected by isolating Siglec-F^+^ splenocytes from IL-5 transgenic mice by fluorescence-activated cell sorting (FACS) on MoFlo XDP or FACS Aria cell sorters maintained by the Research Flow Cytometry Core at Cincinnati Children’s Hospital. Cultured eosinophils (day 15–16 LDBM) and sorted native eosinophils were subjected to centrifugation at 300×g for 5 minutes, washed in 25 mL 1X PBS, and centrifuged again at 300×g for 5 minutes. Cells were resuspended at a concentration of 1×10^6^ per mL in 10 mL 1X PBS and incubated at 37°C for 10 minutes. Cells (100 µL) were collected in triplicate at baseline and after stimulation with 100 ng per mL CCL11 for 10, 20, 30, 60, and 300 seconds. Upon collection, cells were transferred to a 96-well, V-bottom dish, with each well containing 100 µL of 4% paraformaldehyde/1X PBS, and fixed for 15 minutes at room temperature. Cells were pelleted by centrifugation at 500×g for 5 minutes at room temperature and then washed with 100 µL of 1X PBS containing 1% BSA. Centrifugation (500×g, 5 minutes, room temperature) was repeated, and cells were permeabilized with 100 µL of 1X PBS/1% BSA/0.05% Triton X-100 for 15 minutes. An equal volume of 1X PBS/1% BSA solution was added to each well, and centrifugation (500×g, 5 minutes, room temperature) was repeated. Cells were resuspended in 100 µL of 1X PBS/1% BSA solution containing Phalloidin-AF488 (Molecular Probes) at a 1∶1000 dilution and incubated at room temperature for 15 minutes. An equal volume of 1X PBS/1% BSA solution was added to each well, and centrifugation (500×g, 5 minutes, room temperature) was repeated. Cells were resuspended in 1X PBS/1% BSA/0.5% paraformaldehyde solution, and geometric mean fluorescence intensity was determined by flow cytometry.

### Statistics

Data were analyzed using either a two-tailed, unpaired Student's *t* test or a one-way ANOVA with Bonferroni post-hoc test as appropriate (GraphPad Prism). Differences were considered statistically significant when *P*<0.05. Data are presented as mean ± standard error of the mean (SEM), except for representative experiments, in which data are presented as mean ± standard deviation (SD).

## Results and Discussion

### EoPs are a rare cell population in murine bone marrow

**Figure 1:**
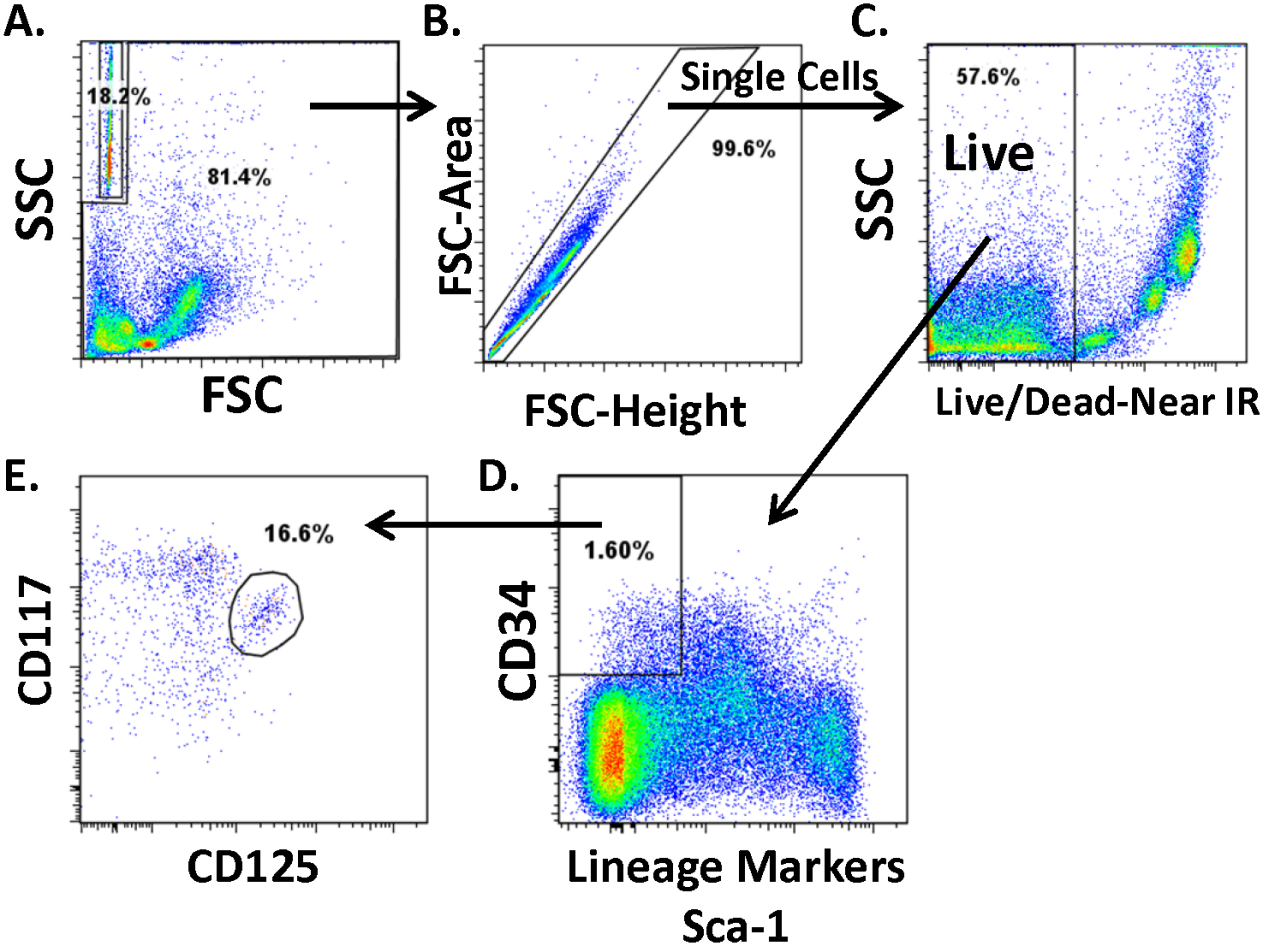
EoP identification via surface markers. Analysis of BALB/c murine whole bone marrow (WBM) by flow cytometry reveals an eosinophil lineage–committed progenitor (EoP) population; gating strategy to delineate EoPs from WBM starts with (A) excluding counting beads (upper left corner) that were added to the cell suspension and then including (B) only single cells that are (C) viable, (D) express CD34 but are negative for lineage markers (CD3, CD4, CD8, CD19, CD45R, Gr-1) and Sca-1 and (E) co-express CD117 (c-Kit) and CD125 (IL-5Rα). All gate frequencies are expressed as a percentage of the parent gate, except for panel A, which shows percentage within that gate. CD, cluster of differentiation; FSC, forward scatter; IR, infrared; Sca-1, stem cell antigen 1; SSC, side scatter.

We first evaluated the number of EoPs in the bone marrow of wild-type BALB/c mice, identifying EoPs by surface marker expression via flow cytometry [10]. To delineate the murine EoP, we initially gated on single, live bone marrow cells that expressed CD34 but were negative for lineage markers (CD3, CD4, CD8, CD19, CD45R, Gr-1) and stem cell antigen-1 (Sca-1) and further gated on cells that expressed both CD125 (IL-5Rα) and CD117 (c-Kit) (Fig. 1). Murine BALB/c EoPs are a rare population comprising 0.132±0.016% of live WBM cells (mean ± SEM, *n* = 6 independent experiments) or 983±253 EoPs per million live WBM cells (mean ± SEM, *n* = 3 independent experiments). Purifying the isolated murine EoPs via cell sorting with stringent gating yielded 319±30 cells per million WBM cells sorted (mean ± SEM, *n* = 4 independent experiments).

### SCF is necessary for optimal eosinophil yield

**Figure 2:**
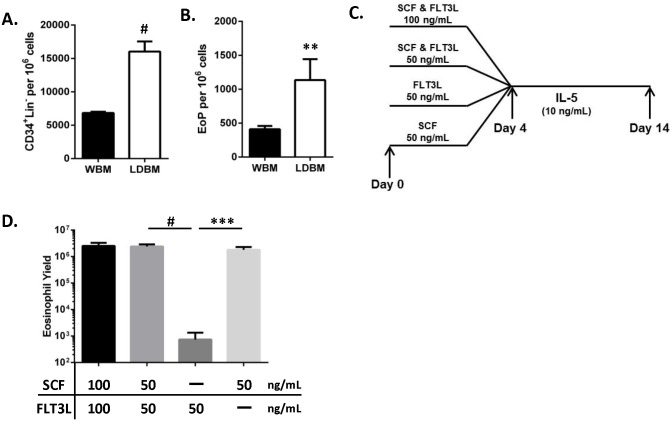
SCF stimulation of LDBM produces optimal eosinophil yield. (A–B) Frequency of (A) progenitor cells that express CD34 but are negative for lineage markers (CD34^+^Lin^−^) and (B) eosinophil lineage–committed progenitors (EoPs) in the cell preparations cultured from whole bone marrow (WBM, black bars) or low-density bone marrow (LDBM, white bars) cells are shown (mean ± SD, representative of 2 independent experiments is shown, *n* = 3–4 mice per group). **P<0.01, ^#^ *P*<0.0001 vs. WBM. (C) Schematic of LDBM stimulation conditions tested prior to IL-5–mediated differentiation is shown; for SCF and FLT3L co-stimulation, the indicated concentration is for each factor individually. (D) Eosinophil yield (mean ± SD) at Day 14 after LDBM stimulation with indicated cytokines followed by 10 days of interleukin 5 (IL-5) stimulation (representative of 3 independent experiments is shown, *n* = 3–4 wells per condition per experiment). ***P<0.001, ^#^ *P*<0.0001. CD, cluster of differentiation; FLT3L, fms-like tyrosine kinase 3 ligand; SCF, stem cell factor.

A previously reported high-yield culture system used WBM to yield phenotypically mature eosinophils [20]. We modified this protocol to start with progenitor-enriched LDBM cells generated by fractionating WBM by density centrifugation (26±3×10^6^ LDBM cells yielded per BALB/c mouse, *n* = 11 independent experiments with 3–4 mice per experiment). LDBM cells contained greater than 2 fold CD34^+^Lin^−^ progenitors and 2.6 fold EoPs compared to WBM cells (Fig. 2A–B), confirming progenitor enrichment of WBM with density centrifugation. In our prior studies [28]–[30], this modified culture system used the cytokines SCF and FLT3L in the first four days of bone marrow culture to increase the number of progenitors prior to IL-5 stimulation. In this study, we evaluated whether both cytokines were necessary for optimal eosinophil yield from LDBM cells in our culture system (Fig. 2C) and observed that stimulating LDBM cells with SCF was necessary and sufficient for optimal eosinophil yield (3.5±0.9 eosinophils [mean ± SD] per LDBM) (Fig. 2D). There was no significant effect on eosinophil yield with the addition of FLT3L to SCF-treated cultures (Fig. 2D).

### LDBM system yields functionally competent eosinophils

**Figure 3:**
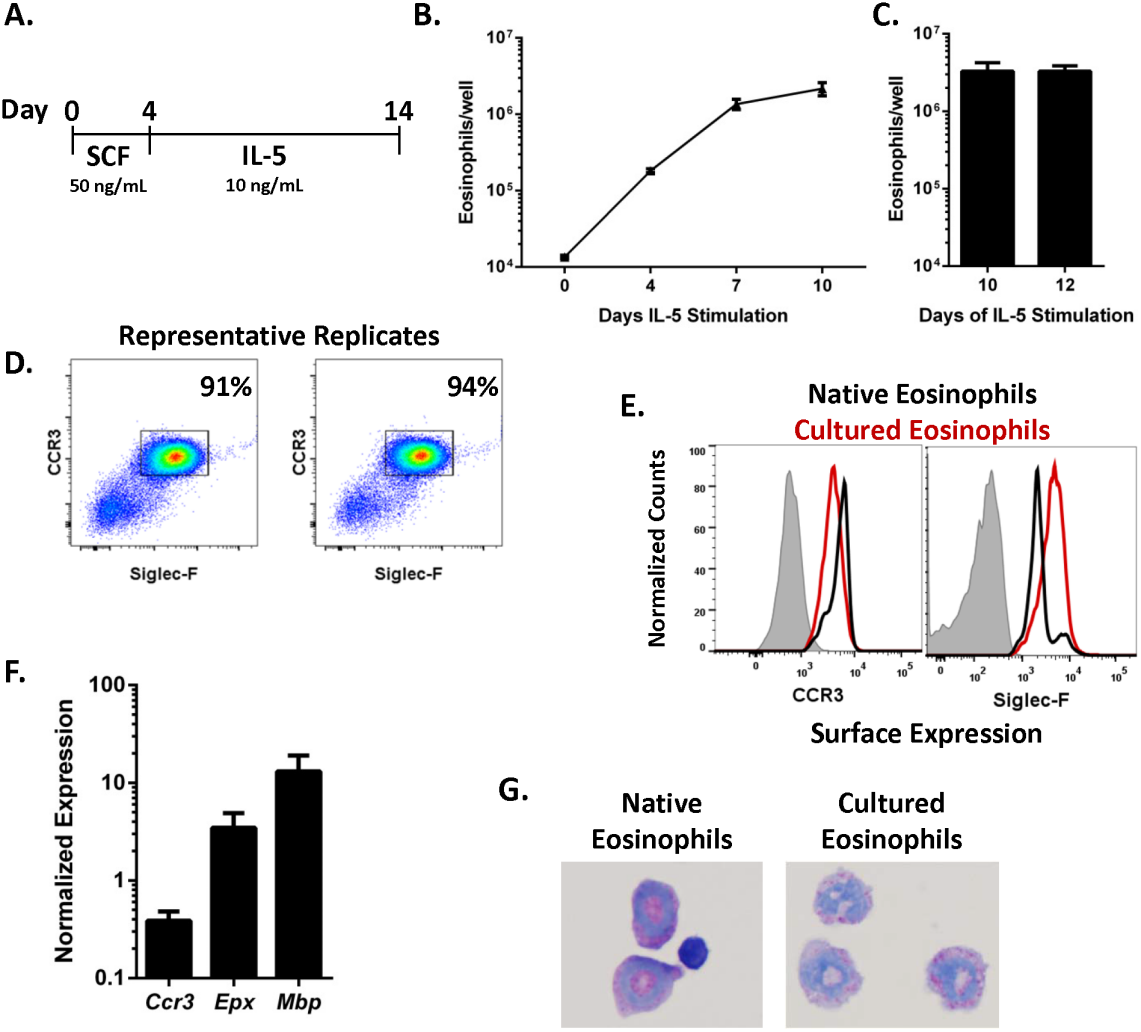
Modified LDBM culture yields mature eosinophils. (A) Schematic of liquid culture for eosinophils is shown. (B–C) Absolute number (mean ± SD, *n* = 3 wells per time point, representative of 3 independent experiments is shown) of eosinophils (CCR3^+^Siglec-F^+^ cells) per well after indicated days of IL-5 stimulation is shown (Days 4–14 of culture schematic presented in panel A and extended to Day 16). (D) Two representative dot blots of Siglec-F and CCR3 expression on the surface of cultured cells at Day 14 are shown with percentage of gated live cells in the upper right corner. (E) Surface expression of CCR3 and Siglec-F by cultured eosinophils (red line) and native eosinophils (black line) compared to isotype control (shaded) is shown. Results are representative of 2 independent experiments. (F) Normalized gene expression (mean ± SD, n = 2 independent experiments) in cultured eosinophils at Day 14 is shown. (G) Light microscopic images of representative cytospin preparations from native eosinophils from naïve bone marrow and cultured eosinophils stained with a modified Giemsa protocol at Day 15. Magnification of 1000X. *Ccr3*, C-C chemokine receptor type 3; *Epx*, eosinophil peroxidase; IL-5, interleukin 5; *Mbp*, major basic protein; SCF, stem cell factor; Siglec-F, sialic acid–binding immunoglobulin-like lectin F.

Our progenitor-enriched eosinophil liquid-culture method (Fig. 3A) yields eosinophils, identified by co-expression of CCR3 and Siglec-F, after SCF and subsequent IL-5 stimulation of LDBM cells. Absolute eosinophil numbers increased linearly within 4 days of IL-5 stimulation and reached a peak absolute count after 7–10 days of IL-5 stimulation (Fig. 3B). No significant increase in eosinophils was noted after 12 days of IL-5 stimulation (Fig. 3C). At Day 14, more than 90% of the cells in the cultures were mature eosinophils (Siglec-F^+^CCR3^+^, Fig. 3D). We next compared expression of known eosinophil surface markers between cultured eosinophils and native eosinophils. Robust surface expression of CCR3 and Siglec-F were detected on both cultured eosinophils and native eosinophils, with a modest decrease in CCR3 and increase in Siglec-F demonstrated on the cultured eosinophils (Fig. 3E). The cultured eosinophils expressed high levels of mRNA for eosinophil-associated genes, including *Ccr3*, eosinophil peroxidase (*Epx*) and major basic protein (*Mbp*, Fig. 3F). The progenitor-enriched culture system yielded eosinophils with a similar Giemsa-stained phenotype to native eosinophils from wild-type murine bone marrow, although granule density appeared to be less in the cultured eosinophils (Fig. 3G).

**Figure 4:**
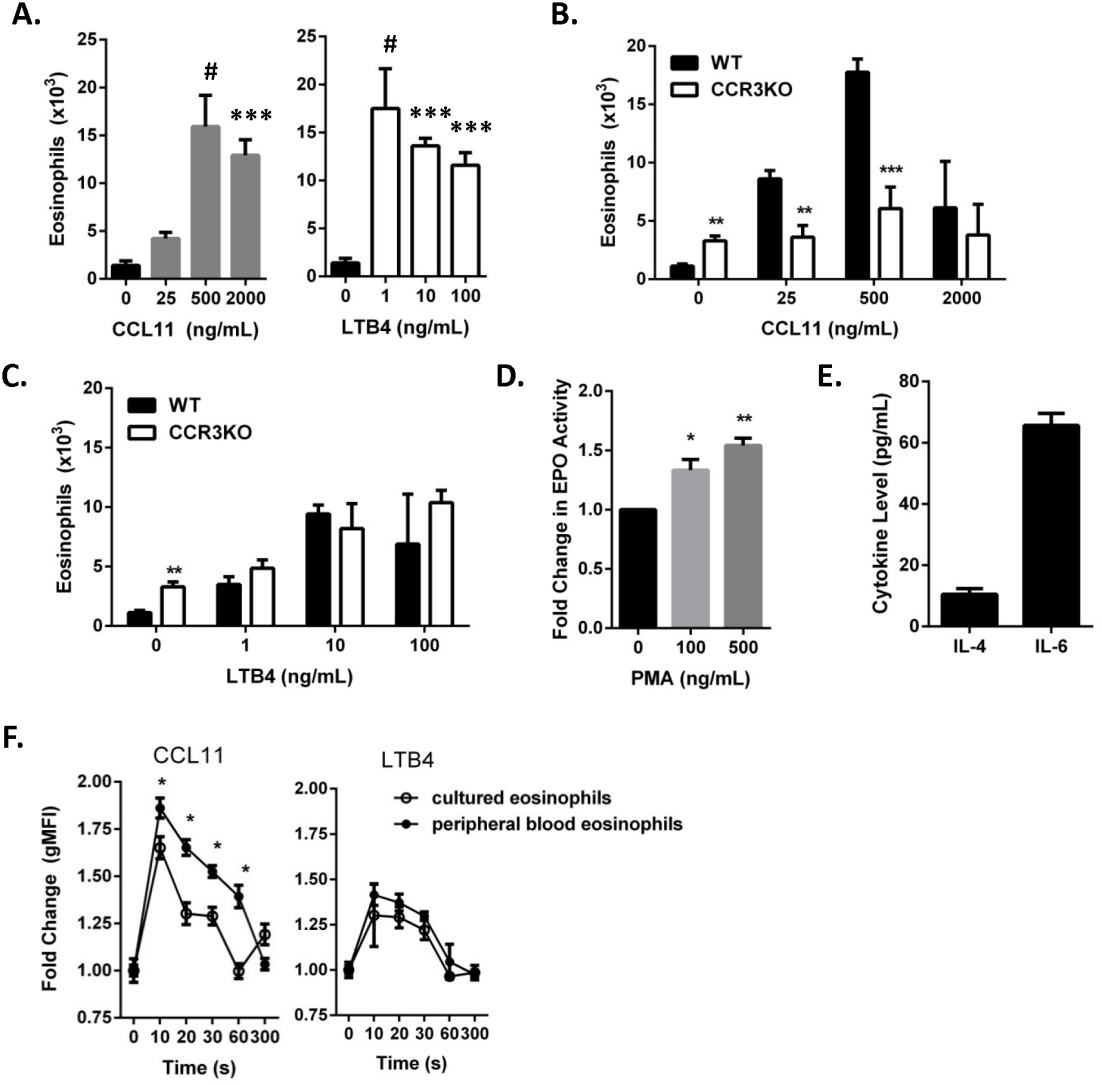
Modified LDBM culture yields functional eosinophils. (A) Total number of cultured cells (mean ± SD, *n* = 3 wells per condition) at Day 14 that migrated toward CCL11 (left panel, gray bars) or leukotriene B4 (LTB4, right panel, white bars) at the indicated doses is shown from a representative of 3 independent experiments. ***P<0.001, ^#^ *P*<0.0001 vs. cells that migrated to media alone. (B–C) Total number of wild-type (WT) and CCR3-deficient (CCR3KO) cultured cells (mean ± SD, *n* = 3 wells per condition) at Day 14 that migrated toward (B) CCL11 or (C) LTB4 at the indicated doses is shown from a representative of 2 independent experiments. **P<0.01, ***P<0.001 vs. WT. (D) Fold change (mean ± SEM, *n* = 4 independent experiments with 3 wells per condition per experiment) in eosinophil peroxidase (EPO) activity detected in the supernatants after activation of eosinophils with phorbol 12-myristate 13-acetate (PMA) at the indicated doses. *P<0.05, **P<0.01 vs. unstimulated controls. (E) Cytokine levels (mean ± SD, representative of 3 independent experiments with 3 wells per experiment shown) in supernatant from Day 14 cells after 24 hours in culture. (F) Fold change (mean ± SD) in total polymerized actin in cultured eosinophils and peripheral blood eosinophils after stimulation with CCL11 or leukotriene B4 (LTB4) at the indicated time points (*P<0.05 vs. cultured eosinophils, representative of 2 independent experiments is shown with *n* = 3 wells per condition and per time point). CCR3, C-C chemokine receptor type 3; gMFI, geometric mean fluorescence intensity; IL, interleukin.

We next evaluated the functional responses of the eosinophils cultured with our modified LDBM culture system. Mature cultured eosinophils displayed a robust chemotactic response toward CCL11 (ligand for CCR3) and leukotriene B4 (LTB4) in a dose-dependent manner with a plateau at the higher doses (Fig. 4A). Eosinophils cultured from CCR3-deficient LDBM cells did not migrate toward CCL11 above baseline, but did migrate toward LTB4, suggesting that the CCL11-stimulated chemotactic response was dependent upon CCR3 and that the cultured eosinophils migrated in an agonist-specific manner (Fig. 4B–C). In addition, activated mature cultured eosinophils degranulated and released eosinophil peroxidase (EPO) into the culture supernatant (Fig. 4D). Eosinophils are known to spontaneously secrete several cytokines [20], [31], and the cultured eosinophils secreted the eosinophil-associated cytokines IL-4 and IL-6 (Fig. 4E). Finally, CCL11 stimulation resulted in actin polymerization in cultured eosinophils and peripheral blood eosinophils, albeit with a modest reduction in the cultured eosinophils but with no differences noted after activation with LTB4 (Fig. 4F). Collectively, these findings indicate that the cultured eosinophils are functionally competent with mild differences in response to mediator activation compared to peripheral blood eosinophils.

### Frozen LDBM cells yield mature eosinophils

**Figure 5:**
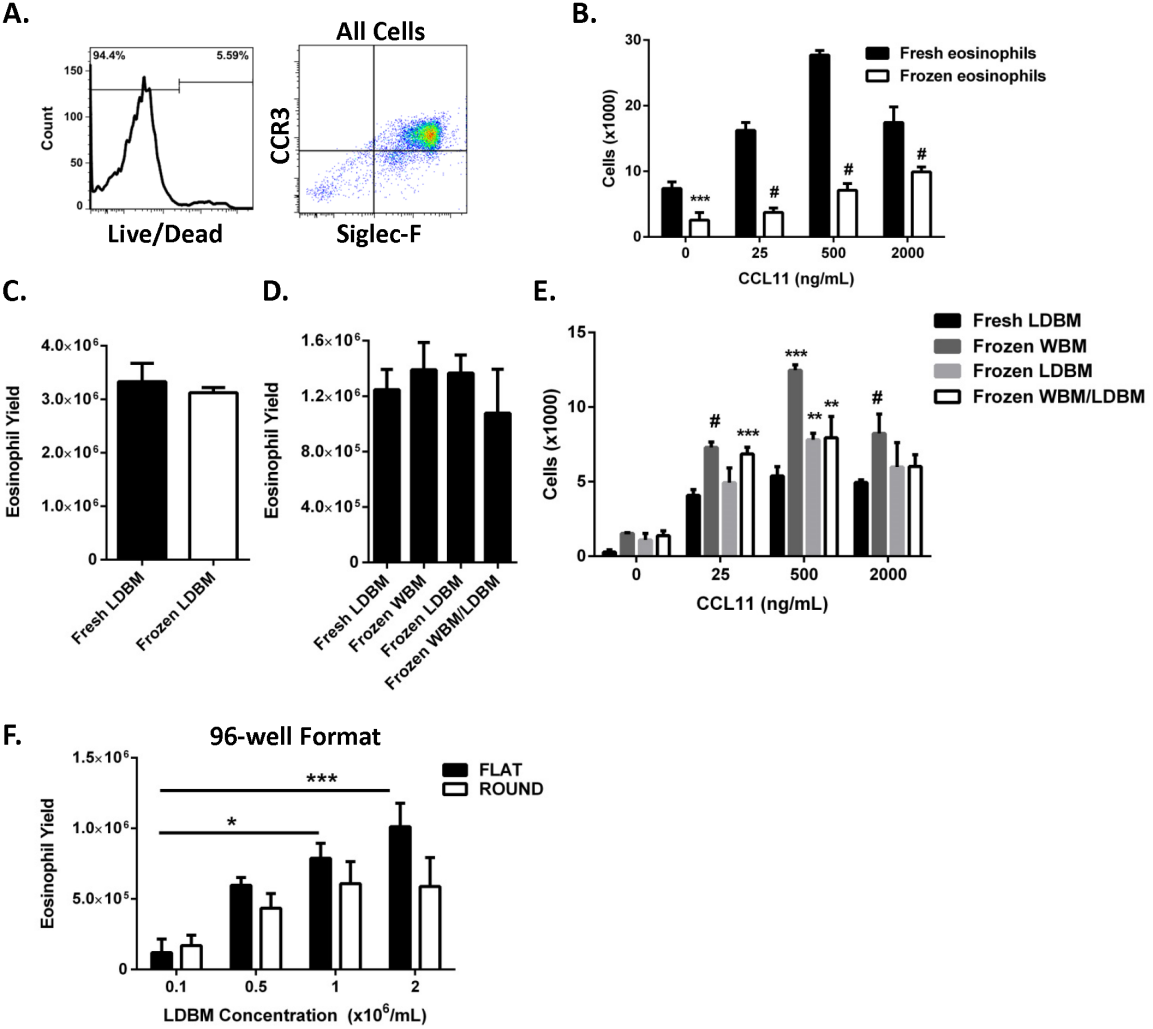
Eosinophils can be cultured from frozen bone marrow cells and in a 96-well plate. (A) Histogram (left panel) of cultured eosinophils that had been frozen, thawed and then stained with viability dye is shown. Percentages of viable (negative for the dye) and dead (positive for the dye) eosinophils recovered from the frozen vial are indicated in the histogram. Surface expression of CCR3 and Siglec-F (right panel) on all cells recovered from the frozen vial is shown in a representative density plot from 4 independent experiments. (B) Total number of (mean ± SD, *n* = 3 wells per condition) Day 15 eosinophils (Fresh eosinophils) or Day 14 eosinophils that had been frozen and then thawed the next day (Frozen eosinophils) that migrated toward CCL11 at the indicated doses (a representative of 2 independent experiments is shown). ***P<0.001, ^#^ *P*<0.0001 vs. fresh eosinophils of the same treatment group. (C) Eosinophil yield (mean ± SEM, *n* = 3 independent experiments with 3 wells per condition per experiment) at Day 14 from cultures started with freshly harvested low-density bone marrow (Fresh LDBM, black bar) or frozen/thawed LDBM (Frozen LDBM, white bar). (D) Eosinophil yield (mean ± SD) at Day 14 from cultures started with LDBM that was freshly prepared (Fresh LDBM), was prepared from whole bone marrow (WBM) that had been frozen and thawed (Frozen WBM), was prepared from WBM and then frozen and thawed (Frozen LDBM), and was prepared from freeze-thawed WBM and then frozen and thawed again (Frozen WBM/LDBM) is shown. Data are a representative of 2 independent experiments with 3 wells per condition per experiment. (E) Total number of eosinophils (mean ± SD, *n* = 3 wells per condition) that migrated toward CCL11 at the indicated doses (a representative of 2 independent experiments is shown). **P<0.01, ***P<0.001, ^#^ *P*<0.0001 when compared to eosinophils from Fresh LDBM of the same treatment group. (F) Eosinophil yield (mean ± SEM) at Day 14 in flat- or round-bottomed, 96-well plates from cultures started with LDBM cells at the indicated concentrations (*n* = 3 independent experiments with 3 wells per condition per experiment). *P<0.05, ***P<0.001. CCL11, C-C motif chemokine 11; CCR3, C-C chemokine receptor type 3; Siglec-F, sialic acid–binding immunoglobulin-like lectin F.

We investigated whether mature cultured eosinophils that were frozen and thawed yielded cells suitable for flow cytometry, as we wished to establish a supply of cells for staining controls. After thawing, mature eosinophils that had been frozen in 90% FBS and 10% DMSO yielded viable cells (94±3% viability, mean ± SD, *n* = 2 independent experiments) for flow cytometry (Fig. 5A). Mature eosinophils (Day 15) that had been frozen and thawed the next day maintained chemotactic activity toward CCL11 but had diminished cell movement compared to eosinophils that had never been frozen (Fig. 5B). Notably, the chemotactic index (number of cells that migrated toward CCL11 at 2000 ng per mL over the baseline of no CCL11) was higher for the frozen/thawed eosinophils than for the eosinophils that had never been frozen (3.8±0.3 vs. 2.3±0.3 [mean ± SD], respectively; *P* = 0.004, n = 3 wells per condition in a representative of two experiments), adding further support for intact functional responses in the frozen/thawed eosinophils.

We next determined whether frozen LDBM cells could be thawed and cultured to generate mature eosinophils. LDBM cells that were frozen in 90% FBS and 10% DMSO (Frozen LDBM), thawed and cultured using the modified protocol yielded similar numbers of mature eosinophils as eosinophils cultured from fresh LDBM cells (Fig. 5C). We also tested the efficiency of WBM cells that were frozen, thawed and then subjected to centrifugation fractionation for LDBM cells (Frozen WBM) prior to plating, as well as whether freezing and thawing of both the WBM and the resulting LDBM (frozen and thawed after both the WBM and the LDBM preparations, Frozen WBM/LDBM) prior to plating affects the yield or function of the eosinophils ultimately cultured from these bone marrow cells. Eosinophil yield was unaffected by freezing/thawing of LDBM alone or WBM alone or freezing/thawing of both WBM and the resulting LDBM (Fig. 5D). Chemotactic activity toward CCL11 was not diminished, but instead enhanced, toward CCL11 for eosinophils whose precursor bone marrow cells had been frozen as WBM, LDBM or both (Fig. 5E).

### Eosinophil culture system can be performed in a 96-well format

To enable culturing with a small starting population, we determined the optimal number of LDBM cells that could be plated in either a flat-bottom or round-bottom, 96-well tissue culture plate to yield the highest number of mature eosinophils. Eosinophil yield was dependent upon the number of LDBM cells plated in the flat-bottom wells and, to a lesser extent, in the round-bottom wells (Fig. 5F).

Differentiation of EoPs to mature eosinophils is a multistage process requiring the coordinated activity of multiple regulators. However, the developmental program that orchestrates eosinophil maturation is not well delineated. Studies focused on EoP differentiation are inherently difficult to perform due to the rarity of EoPs in the bone marrow under homeostatic conditions; they comprise ∼0.05–0.1% of total murine bone marrow cells [10]. As targeting eosinophil production with lineage-specific blockade has clinical potential as treatment for eosinophilia, robust and novel methods are needed to support and encourage studies into eosinophil development.

In this manuscript, we reveal advances and modifications to the LDBM culture system protocol that was previously described [28]–[30], [32], [33]. We have determined that stimulation of the progenitors with FLT3L is not necessary and that a lower dose of SCF is sufficient to yield mature functional murine eosinophils, which may provide a substantial cost savings to investigators. As SCF was determined to be necessary (and sufficient) for optimal eosinophil yield (3–5 eosinophils per LDBM cell or 5,400 eosinophils per EoP in the LDBM preparation), we believe that a small expansion occurs initially via SCF/c-Kit signaling in the progenitors and precursors in the LDBM preparations and that this small expansion is then followed by robust IL-5–induced expansion of the IL-5Rα–expressing progenitors and precursors. FLT3 signaling has been shown to be important in lymphoid commitment from early hematopoietic progenitors [34], and FLT3L has been utilized to generate dendritic cells in cultures [35]. However, the small number of progenitors within the LDBM preparations that would be responsive to FLT3L (FLT3^+^) are likely not responsive to IL-5 and thus not further expanded in this LDBM culture system.

For research investigations, it is extremely advantageous to be able to freeze bone marrow cells and have them retain viability with the capability of differentiation upon thawing. In the described protocol, we indicate opportunities where cells can be frozen without harmful consequences to experiments (S1 Fig.). Our studies demonstrate that frozen bone marrow cells, both WBM and LDBM, can be used effectively for differentiation into mature eosinophils. Collection and storage of bone marrow can thus be separated from culture preparation by time and geography, greatly decreasing the complexity of factors involved in experimental planning and expediting new experiments. Further, we demonstrate that cultured mature eosinophils can be frozen and thawed for use in functional studies and as viable reference control cells for flow cytometry. This precludes the necessity of isolating fresh eosinophils for controls for complicated flow cytometry panels when the experimental groups have low numbers.

The expense of liquid cultures rises dramatically with cytokine use and volume of cultures. As cytokine use is inherent in the study of eosinophil differentiation, we successfully scaled down our protocol to culture LDBM cells in a 96-well format. Keeping the volumes low in a 96-well format without sacrificing effective EoP and eosinophil precursor differentiation allows for increased numbers of wells available for replicates and conditions to be tested, including high-throughput screens of drugs and small molecules that target EoPs. In addition, using a 96-well format requires fewer bone marrow cells, enabling EoP differentiation studies with rare strains of mice.

Herein, we describe a modified progenitor-enriched culture system (S1 Fig.) that represents a new, powerful tool for murine eosinophil research and provides significant adaptability in experimental design, including the scale and cost of liquid cultures.

## Supporting Information

S1 Fig **Eosinophil liquid-culture system developed for EoP enrichment and adaptable experimental design.** Whole bone marrow cells can be harvested from the long bones of mice and frozen at −80°C for future studies or subjected directly to density centrifugation to separate progenitor-enriched low-density bone marrow (LDBM) cells. The LDBM cells can then be frozen at −80°C for future studies or transferred immediately to tissue culture plates (96-well, 24-well or 6-well plates) containing culture media with added SCF (50 ng per mL) for 4 days followed by differentiation in media containing IL-5 (10 ng per mL) for 10 days. Mature eosinophils can then be evaluated for maturation status or frozen at −80°C and thawed for future studies. IL, interleukin; SCF, stem cell factor.
(TIF)
